# Exploring Optimization of Zeolites as Adsorbents for Rare Earth Elements in Continuous Flow by Machine Learning Techniques

**DOI:** 10.3390/molecules28247964

**Published:** 2023-12-06

**Authors:** Óscar Barros, Pier Parpot, Isabel C. Neves, Teresa Tavares

**Affiliations:** 1 CEB—Centre of Biological Engineering, University of Minho, Campus de Gualtar, 4710-057 Braga, Portugal; parpot@quimica.uminho.pt (P.P.); ttavares@deb.uminho.pt (T.T.); 2 CQUM, Centre of Chemistry, Chemistry Department, University of Minho, Campus de Gualtar, 4710-057 Braga, Portugal; 3 LABBELS—Associate Laboratory, 4710-057 Braga, Portugal

**Keywords:** rare earth elements, zeolites, machine learning, sorption processes, circular economy

## Abstract

Unsupervised machine learning (ML) techniques are applied to the characterization of the adsorption of rare earth elements (REEs) by zeolites in continuous flow. The successful application of principal component analysis (PCA) and K-Means algorithms from ML allowed for a wide range assessment of the adsorption results. This global approach permits the evaluation of the different stages of the sorption cycles and their optimization and improvement. The results from ML are also used for the definition of a regression model to estimate other REEs’ recoveries based on the known values of the tested REEs. Overall, it was possible to remove more than 70% of all REEs from aqueous solutions during the adsorption assays and to recover over 80% of the REEs entrapped on the zeolites using an optimized desorption cycle.

## 1. Introduction

Continuous research and progress have resulted in a significant surge in available data, motivating some sectors of our society to reposition themselves and harness the disruptive potential of data analytics and machine learning [1]. Machine learning (ML) is an evolving branch of computational algorithms, and its development has led to statistical models that can make predictions and support decisions without being explicitly programmed [2,3,4,5]. ML can integrate multimodality multi-fidelity data to reveal correlations between different features [6]. It has been applied successfully in diverse fields such as pattern recognition, medicine, science, computer vision, spacecraft engineering, engineering, biomedicine, psychology, catalysis, neurobiology, and many other disciplines [1,5,7]. This wide application allows for a faster treatment of great amounts of data and, therefore, ML can be used to analyze and correlate those data to achieve better interpretations and, therefore, to make better decisions.

ML models and their importance have been recognized and appreciated in wastewater treatment [8,9]. Some developments have been made to use ML algorithms or deep learning neural networks for the optimization of the adsorption of antibiotics [10,11], organic compounds [12,13], and metals [14,15,16].

The advantages of the ML techniques applied to the recovery of rare earth elements (REE) from aqueous solutions using zeolites as adsorbents are described. REEs represent 19% of the metals used in the technology and precious metals sectors, which accounts for 0.05% of world metal production and the trend is upwards [17,18]. REEs have played a crucial role in the materials industry across various domains such as phosphors, magnets, metallurgy, catalysts, and glass since the 1950s. They are frequently employed as additives or dopants in materials formulations. REEs are particularly valuable due to their ability to induce significant changes in material properties, even when used in small quantities. Consequently, they have earned the reputation of being the “vitamins” of modern industry and the design of materials doped with rare earths has emerged as indispensable for technological advances [18].

Zeolites are porous aluminosilicate materials known for their highly structured crystalline network composed of alumina and silica tetrahedra (TO_4_, where T = Si or Al). The presence of alumina induces a negative charge on the structure that is compensated for by cations. The cation exchange capacity of the zeolites enhances their usage as adsorbents [19]. Within the zeolitic structures available in the commercial market, zeolites of the LTA type and FAU type (*faujasite*, including zeolites X and Y) are commonly applied in various fields [19,20,21]. The FAU structure exhibits a low Si/Al ratio, which results in a high cation exchange capacity mainly for cations with high charge density [19], such as rare earth elements (REE ions). A microporous framework characterizes the FAU structure and to increase its mesoporosity, some modifications are required, which will result into an improvement of the ion exchange capacity of the structure. The modification of zeolites can be achieved by chemical or hydrothermal treatments, which normally lead to an improvement in a zeolite’s porosity [22,23]. The chemical treatments can be performed by the use of mineral salts, alkaline or acidic solutions, which can improve crystal size, morphology and chemical composition to enhance their adsorption capacity [24,25]. The hydrothermal treatment consists of a heat treatment through the contact between the zeolite and an aqueous solution to improve the zeolite ion exchange capacity [26]. In the case of the zeolite structures with a high silica content, such as MFI or BEA, their surface hydrophobicity can be modified by chemical treatments to lead a better accessibility to the active acid sites due to the introduction of a secondary mesoporous network of inter or intracrystalline nature, which improve their adsorption capacities. Also, the MOR structure can be modified to improve their adsorption behavior by desilication [27,28,29]. In this work, the NaX from FAU was modified using the alkali treatment.

The application of ML algorithms has been applied to REE separation techniques [30] and adsorption [31]. Therefore, the objective of this study is to optimize the removal of REEs (La, Eu, Pr, Ce, Tb and Y) by adsorption on FAU structures in continuous flow assays, employing ML techniques for evaluation and system development.

## 2. Results and Discussion

The overall and simultaneous analysis of the results for the adsorption and desorption cycles (Figure S1) revealed the complexity of observing data without noticeable differences. The solution pH was monitored and adjusted when required to avoid the eventual REE precipitation. The same happened for the desorption assays (Figure S2). The sorbent washing with NaOH 0.01 M was performed after cycles 1, 2, and 3 and not after the last cycle. No REE leaching was detected during this procedure. During the analysis, removal will refer to the adsorption results, while the recovery will refer to the desorption ones.

### 2.1. Machine Learning Analysis

The ML analysis of the continuous flow assays was used to evaluate and select the best conditions among the tested ones, as previously described. Before applying the unsupervised ML algorithms, performance of a data scaler is required, which consists of a data normalization.

**Figure 1:**
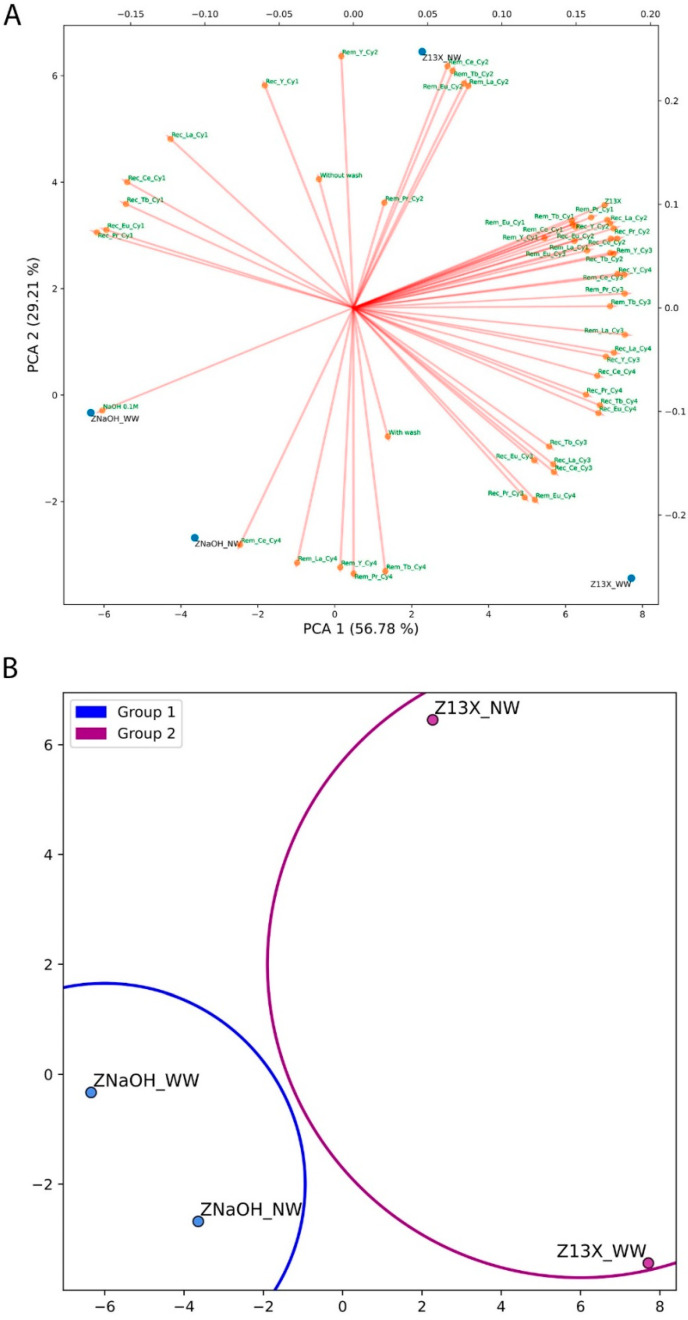
ML analysis of the four conditions used: (**A**) PCA analysis; (**B**) K-Means algorithm. The Rem refers to each REE removal (adsorption), Rec refers to each tested REE recovery (desorption) and the Cy regards each cycle.

The significance of each principal component weight in principal component analysis (PCA) was assessed (Figure S3A) and two features were selected to build the PCA as they justified 86% of the variance. The two features’ selection was not confirmed by the Knee Locator method [32]. The resulting PCA representation is shown in Figure 1A. The PCA was too crowded with features for the evaluation, and it was hard to read the influence of each one on the tested conditions. Nevertheless, the majority of the features seemed to have a high impact on the controls Z13X_NW and Z13X_WW (zeolite 13X, without and with a NaOH 0.01 M washing after the desorption). The PCA analyses, Figure 1A, are represented in a *biplot* where the bottom *x* and left *y* are references for the samples distribution, while the top *x* and right *y* are for the features distribution.

The division using the K-Means algorithm created two different groups, shown in Figure S1. Similar to the PCA, the Knee Locator method did not identify any value for the best number of clusters. In Figure 1B, the four zeolite samples were divided into two groups, one for the pristine zeolite (Z13X_NW and Z13X_WW) and another for the modified Z13X with NaOH 0.1 M (ZNaOH_NW and ZNaOH_WW), without any other division regarding the washing.

The ML classification algorithms were used to assess one of the four tested samples (Table 1). 

Each sample must be classified using a binary system, where a value of 0 indicates poor performance, whereas 1 is indicative of good performance. The binary classification was performed according to Table 2. 

The cut-off for satisfactory results was a classification mean equal to or above 3.5 and none of the 4 tested conditions were classified as 1. The classification means were calculated following Table 2 and the results for the four conditions ranged between 2.00 and 2.50, and since each part (adsorption or desorption) has a possible total weight of 2.5 out of 5.0, it is very likely that one of them underperformed. From overall results of the adsorption (Figure S1) and desorption (Figure S2), it can be concluded that the desorption underperformed with recoveries below 30%, which may explain the obtained results. Somehow this was unexpected as the zeolite 13 X with the NaOH 0.1 M treatment performed much better REE removal and recovery than the Z13X itself. An incomplete recovery may negatively affect subsequent adsorption due to previous occupation of the different sites of the zeolite by the REEs retained during the first adsorption steps.

### 2.2. Sorption Analysis of the Continuous Flow Assays Cycles

#### 2.2.1. Adsorption Analysis

The removal percentages are the results for specific time points of 24, 48, and 72 h and the results are shown in Figure S1 for the different cycles. The results of the removal for the first cycle are similar at each analyzed time point, with no significant differences observed. As expected, the removal values increased over time, confirming REEs adsorption by the zeolite samples. For the second cycle, the total removal shows similar results, with most of the statistical tests having no significant difference.

The same behavior would be expected for the subsequent cycles. The results of total removal for the third and fourth cycles show that no significant differences were found. These results indicate that the ion exchange capacity of the zeolite attains the equilibrium, which is more visible for these cycles than the one obtained for the second cycle since it was used at a higher concentration. The total removal of REEs for each tested condition was calculated and the results are shown in Table 3.

Overall, between 65 and 90% of the total mass of REE present in the solutions to be treated were removed and the zeolite retained similar selectivity between the different tested REEs for each of the four conditions (Table 3). No significant difference was found between the different tested conditions (Table S1), even between the control zeolite, Z13X, and the ZX_NaOH, as foreseen by previous batch assays. This suggests that a pre-treatment of the zeolite will not improve the sorbent behavior.

A difference between the NW and WW (without and with NaOH washing) was expected for both zeolite samples. It was expected that the washed columns (Z13X_WW and ZNaOH_WW) would reach higher removals as the hydroxyl (OH^−^) from the NaOH could neutralize part of the protons (H^+^) from HNO_3_ used in the desorption step. The NaOH concentration used, 0.01 M, was not enough for this purpose since its concentration was 10 times lower than the acid.

#### 2.2.2. Desorption Analysis

The desorption results for the four different cycles are shown in Figure S2. The desorption results for the first cycle are shallow, below 30%, with no significant difference found for the different comparisons evaluated. For the second cycle, the desorption recovered below 11%, with no significant difference being observed. The same occurred for the third cycle, with recoveries below 10%, and the fourth cycle, with recoveries below 14%. No significant difference was found.

These results were not expected since the same concentration was used for the acid chosen to be the best one in batch assay. The same study reported that the NaOH 0.1 M zeolite had a very-high recovery from the tests performed in the batch assays. The low recoveries of REE probably could be related to the saturation of the zeolite structure. Nevertheless, it was not evident for the second cycle due to the lower initial REE concentration of 10 mg/L, supported by the adsorption results (Figure S1). The third and fourth cycles had higher initial REE concentrations of 60 and 25 mg/L, respectively, where the lower removal was more evident, as supported by the adsorption results (Figure S1).

This shows the importance of the desorption step in a multiple-column cycle assay, since good desorption might lead to a near-total removal of REEs from the zeolite. After that and during the second adsorption cycle, the zeolite would be available to recover more REEs from the solution. The total recovery was calculated and shown in Table 4.

The overall desorption results were below 20% of the total adsorbed REE, with no significant differences (Table S2). These results were meager and unexpected since higher recoveries were achieved in batch assays. This raises a suspicion that a higher concentration of the acid should be used in these assays, so the desorption may be improved.

After the desorption process, a purification step will be required to allow for the re-use of the recovered REEs into new applications. The purification process may be the precipitation of the REEs as carbonates [33,34] or oxalates [35,36,37,38,39].

#### 2.2.3. ML Analysis of the Desorption Optimization

**Figure 2:**
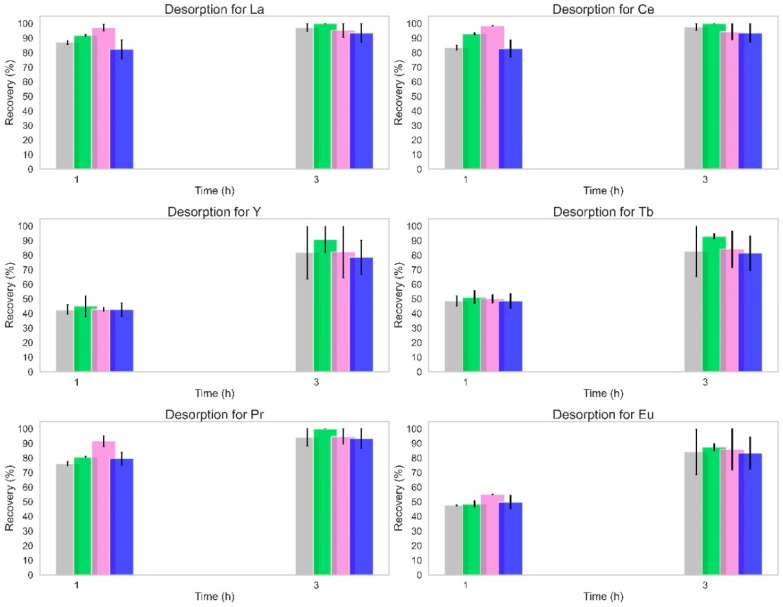
Total recovery for the different REE from loaded Z13X_NW (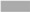), Z13X_WW (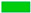), ZNaOH_NW (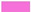), and ZNaOH_WW (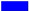) for the 1 M eluent. The NW refers to the assays without the washing step and the WW refers to the assays with the NaOH 0.01 M washing step. Samples were taken from the accumulation Erlenmeyer with the outflow eluent.

In the desorption batch assays, 0.35 g of loaded zeolite was used with 0.1 L of HNO_3_ at 0.1 M, which defined a ratio of 28.6 mmol of HNO_3_ per g of zeolite. In these continuous flow assays, the ratio was 0.67 mmol of HNO_3_ per g of zeolite. The proportion between the batch ratio and the column ratio was 43, which explains the reduced desorption efficiency in continuous flow assays. So, it was decided to perform new desorption assays in the continuous flow set-up with a ratio of 13.3 mmol of HNO_3_ per g of the zeolite (2 L of 1 M of HNO_3_ during 3 h with the same flow rate in close loop). The results obtained with the optimized acid/zeolite ratio are shown in Figure 2.

The changes implemented for the desorption were definitive to improve the REE recoveries as seen in Figure 2. After 1 h of assays, over 70% of La, Ce, and Pr were removed from the zeolite, while the other REE had a lower recovery. This difference could be related to the accessibility of the cations located in the zeolite structure. The structural framework of zeolite Y or X (FAU) were distinguished by three main units: the hexagonal prism, the sodalite cavity, and the supercage [40]. The recovery of these cations is facilitated if they are primarily located in the sites of the supercage or sodalite cavities. The difference between REE radii could justify the observations, since a smaller ion may easily enter deeper into the smaller pores of the zeolite and, therefore, would require more time to desorb from it.

After a 3 h leaching, the recoveries of REE were similar between them and over 80%. It is important to add that no significant differences were found between the tested conditions and that the primary source of variation was the time for all tested REEs. Also, this recovery refers to the total REEs still entrapped in the zeolites after the four cycles of adsorption and desorption, which shows that this optimization could lead to an increase in REE adsorption in following cycles.

The last time point results of all tested desorption cycles (the first four and the one with higher acid concentration) were compared for the same tested conditions. A significant difference, Table S3, was found for all REEs when any cycle was compared with cycle 5 (desorption with 1 M acid). This is validated by Figure 2, as the optimized cycle presented recoveries up to four times higher than the ones from the previous cycles.

A new DataFrame was built just considering the desorption results in order to test the eventual supervised ML analysis. This DataFrame consisted of the original four cycles and the new desorption cycle, with a higher ratio of acid/zeolite, and it was used in a new ML analysis to investigate the impact of the amount of acid on the desorption efficiency.

**Figure 3:**
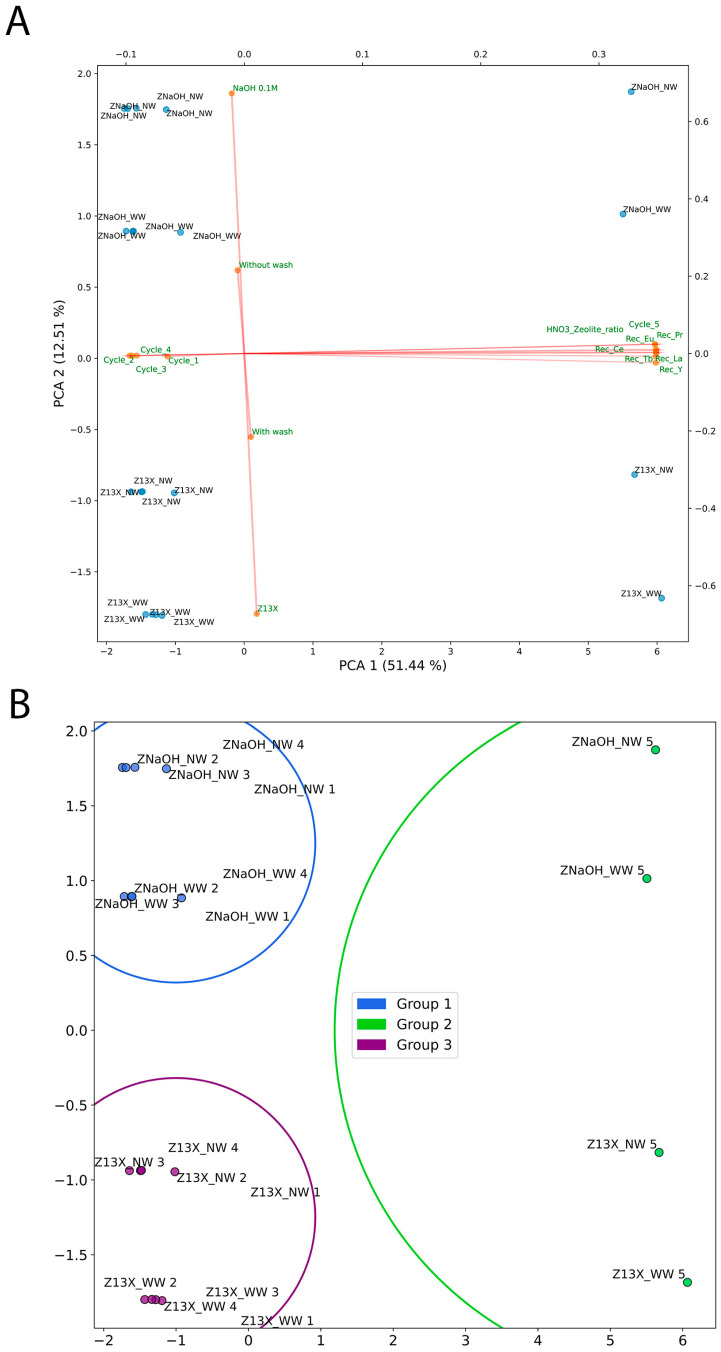
ML analysis: (**A**) PCA; (**B**) K-Means. The Rec refers to the recovery of each tested REE.

From the results in Figure S4A, four components were selected to build the PCA, confirmed by the Knee Locator method [32], and the result is shown in Figure S5. The first two features explain 64% of the variance in the samples, as shown in Figure 3A. The PCA distribution results in two main groups, one on the right side, more influenced by cycle 5 and respective recoveries of the different REEs. The other group, on the left side, is more affected by the first four cycles. This group can be divided into two smaller groups: the top related to action of the ZX_NaOH zeolite and the bottom for Z13X. Each zeolite group can be further divided into two groups depending on the washing after cycles, with NW in the top and WW in the bottom.

The K-Means were made using three groups, as shown in Figure S4B, confirmed by the Knee Locator method [32]. A clear group 1, in blue, which was from the ZX_NaOH zeolite for both NW and WW, can be seen in Figure 3B. The group 2, in green, was affected by the fifth cycle of desorption with the results of both ZX_NaOH and Z13X, and group 3, in purple, is the Z13X zeolite. The other groups are mixed.

**Figure 4:**
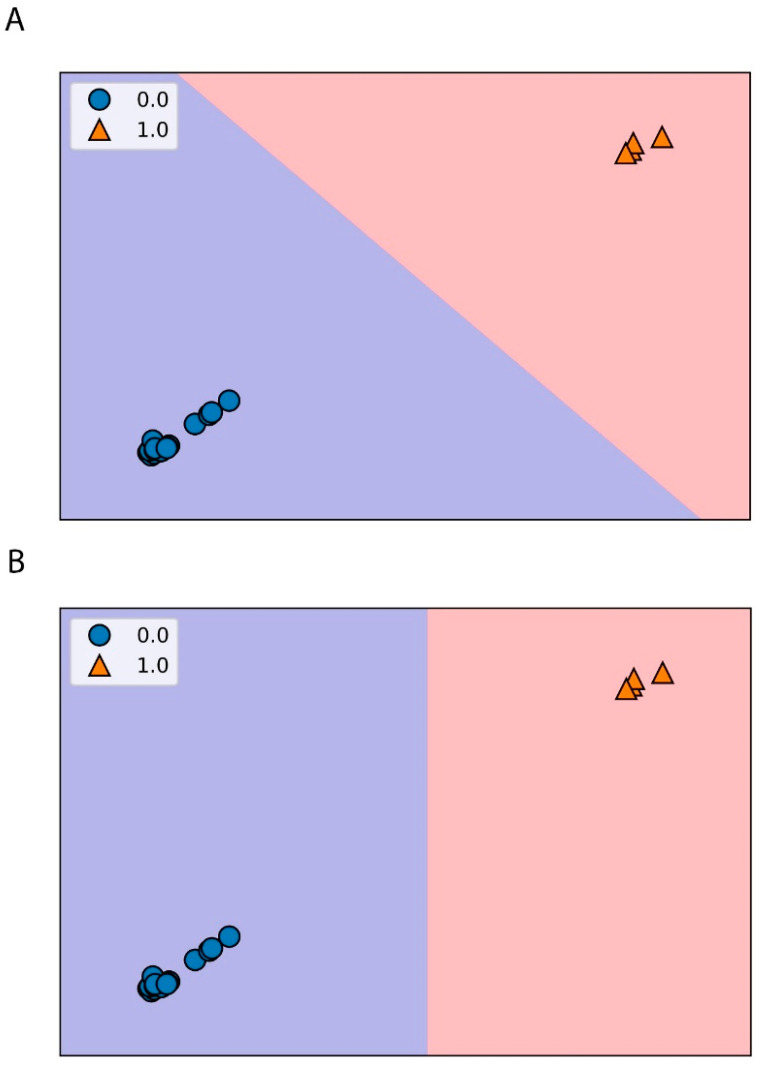
Conditions division according to the classifiers: (**A**) KNN and logistic regression and (**B**) decision tree and random forest. The 1 represents a good desorption, while the 0 is a bad desorption according to the evaluation performed. The different colors, violet and orange, represent the zone of a good or bad sorbent, respectively.

The ML classification algorithms were used to select the best desorption conditions, using the binary classification (Table 2). For this case, four samples were considered good according to Table 2. The four selected samples are the ones from the cycle 5, as expected, since this cycle was the best one. With this, the classification was carried out using four classifiers, KNN, Decision Tree, Random Forest, and Logistic Regression. The results of the different classifiers are shown in Figure 4. The use of these classifiers is due to their wide application as learning algorithms [41,42,43]. The KNN, Decision Tree, Random Forest, and Logistic Regression classifiers are simple, easy to implement, and versatile [41]. At the same time, these algorithms are more adequate for use with a relatively small number of data and their hyperparameters can be more easily optimized [41,42,43].

For the KNN, Figure 4A, three neighbors were selected according to the accuracy values for both training and test sets (Figure S4C). The Decision Tree classifier, Figure 4B, has only one parameter, random state, with a value of 20. For the Random Forest, Figure 4B, the n estimator parameter was 10 and the random state was the same as for the Decision Tree classifier. Finally, the same value for the random state parameter was used for the Logic Regression, Figure 4A.

It is crucial to avoid overfitting of the training set for the classifiers, as happens often with the model. A suitable generalization of the model from the training set can lead to a good classification of new and unseen data, which is the test set. All the tested classifiers could separate the two groups without any problem. Therefore, it is expected that the respective accuracy scores of the values (x and y values of training and test data) would be 100%. The scores of the training and test using the four different classification algorithms were 100% for all tested classifiers. Similar results were obtained using the classification report, which summarizes percentages of precision, recall, and f1-scores. It is important to know that precision is related to the accuracy of making good predictions, recall is the value of the correctly identified positive predictions, and f1-score is the harmonic mean of the precision and recall. This evaluation used the real classification from the binary classification (*y_real*) and the predicted classification (*y_pred*) after training the model.

All classifiers presented a 100% score for the precision, recall, and f1-scores of the prediction the model, considering the real classification of 100% for each one. Another vital metric to assess the classification used is the confusion matrix. All the classifiers evaluated were very similar between them and the overall result is shown in Figure S6. It was verified that there were only true positives (the model predicted it was a cycle with high desorption, and it was in fact high) and true negatives (the model predicted it was a cycle with low desorption, and it was actually low).

**Figure 5:**
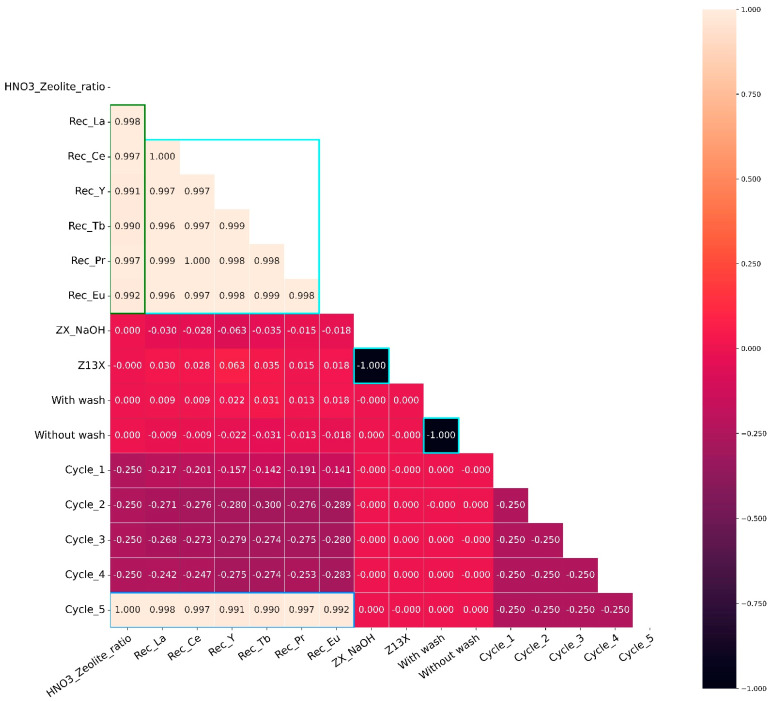
Heatmap representation of the correlation of the features used for the desorption obtained in the different cycles. The results for the maximization of the desorption are referenced as cycle 5. The left scale represents the different correlation values and the respective colors.

A heatmap showing the Pearson correlations of the tested features was made as before, and the results are shown in Figure 5.

Overall, the correlation for the desorption cycles can be considered negligible, with three main exceptions. The first relates to the zeolites (Z13X and ZX_NaOH) and to the washing (NW and WW), which have a very high negative correlation, as shown in Figure 5. In addition, the correlation of the recovery of each REE shows a very-high positive, which is expected since all REEs had a higher recovery. Finally, as expected, cycle 5 shows a very-high positive correlation with the REE recoveries since it was the cycle with higher REE recoveries. The same happens for the HNO_3_/zeolite ratio correlation with the REE recoveries for the same reason as stated before. Also, this ratio has a very high positive correlation with cycle 5 as expected, since the highest HNO_3_/zeolite ratio was used in this cycle.

## 3. Materials and Methods

### 3.1. Materials

The REEs were used as salts: europium (EuCl_3_·6H_2_O; 99.9%, Acros Organics (Geel, Belgium)), cerium (Ce(NO_3_)_3_·6H_2_O; 99.5%, Acros Organics), lanthanum (La(NO_3_)_3_·6H_2_O; 99.9%, Alfa Aesar (Kandel, Germany)), praseodymium (PrCl_3_·xH_2_O; 99.9%, Alfa Aesar), terbium (TbCl_3_·6H_2_O; 99.9%, Alfa Aesar), and yttrium (YCl_3_·xH_2_O; 99.9%, Alfa Aesar). These metal salts were used from previously prepared stock solution at 2000 mg/L. The multi-element ICP quality control standard solution, with a concentration of 200 mg/L of each element, was purchased from CPAchem (Stare Zagore, Bulgaria). The zeolite structure FAU (Z13X), used in pellet form, was supplied from Acros Organics. The particle size for Z13X beads ranged from 4 to 8 mesh with an average pore size of 7.4 Å and with a Si/Al ratio of 1.50.

### 3.2. Analytical Quantification of REE

All liquid samples were analyzed at the Inductively Coupled Plasma-Optical Emission Spectrometry, ICP-OES, (Optima 8000, PerkinElmer, Shelton, CT, USA). The procedure was very similar to the one reported by Barros et al. [44], where the liquid sample was filtered through a pore size of 0.22 µm and some drops of nitric acid, HNO_3_ (Fisher, Loughborough, UK, 69%), were added to avoid a pKa value change. This analysis was performed with slightly different operating conditions, namely, RF power at 1400 W, argon plasma flow at 12 L/min, auxiliary gas flow at 0.2 L/min, and nebulizer gas flow at 0.70 L/min. The wavelengths (nm) used for each element were: La—408.672, Ce—413.764, Eu—381.967, Y—371.029, Tb—350.917, and Pr—390.844, with an axial plasma view for La, Ce, Tb, and Pr, while for Y and Eu, a radial view was used.

### 3.3. Continuous Flow Assays

The continuous flow assays were carried out using 150 g of zeolite with and without surface modifications as the bed of column (height of 30 cm and diameter of 4.2 cm) and set-ups with up-flow feeding. Briefly, the zeolite modifications consisted of of a wash with deionized water (dH_2_O) for 6 h with a flow rate of 23 mL/min followed by a wash with NaOH 0.1 M for 22 h with a flow rate of 3 mL/min. The modified zeolite is identified as ZX_NaOH, while the zeolite without modification as Z13X. The column designations are listed in Table 1.

Each column was run in 4 cycles, which consisted of an adsorption and a desorption step. Two of the four columns (ZX_WW and ZNaOH_WW, Table 1) had a washing step between the desorption and the following adsorption, which was repeated 3 times. The cycle steps description with concentrations, pump rate, and duration is presented in Table 5.

The adsorption assays were carried out using a solution with the six different REEs: La, Eu, Pr, Ce, Y, and Tb. Cycles 1 and 3 were run with a 60 mg/L solution of each of REE, while the initial concentration of each REE was 10 and 25 mg/L for cycles 2 and 4, respectively. Samples from the feeding solution in the retention Erlenmeyer were also taken at 0, 24, 48, and 72 h, and the measured concentrations were used to define the function of the removal (%) versus time. At the end of the adsorption step, the REE solution was drained from the columns and afterwards the desorption solution, HNO_3_ at 0.10 M, was introduced. Samples were taken from the solution in the retention Erlenmeyer at 0, 1, 2, 4, and 6 h. Finally, in the last phase, NaOH 0.01 M was used for pH equilibration and washing. This step was performed in just one column of each zeolite, namely Z13X_WW and ZNaOH_WW, with samples taken from the solution in the retention Erlenmeyer at 0 h and after 2 h.

### 3.4. Machine Learning

The DataFrame, a table where the rows list the samples used while the columns are the different parameters used to evaluate the samples, supported the machine learning (ML) algorithms.

The first ML evaluation objective was the selection of the most suitable tested condition (considering the zeolite and the washing option). For that, the 4 different conditions tested (Table 1) were listed as rows, with 50 characteristics (24 adsorption and 24 desorption results: considering 4 different samples and 6 REE, plus the zeolite used and the eventual washing between adsorption and desorption), which were mainly the results of the adsorption (removal) and of the desorption (recovery) for each REE tested in each cycle.

The second ML evaluation was meant to validate any good cycle regarding the removal and recovery of the different REE. For that, 16 samples were used (the previous 4 samples were divided according to the 4 cycles of adsorption and desorption for each REE) and 15 columns with the respective results (6 removals and 6 recoveries for each REE, the cycle number, the zeolite used, and the eventual washing between adsorption and desorption) were used to assist in the selection of the best cycles.

The DataFrame was processed under an unsupervised learner (principal component analysis, K-Means analysis) and a supervised learner (classification). Briefly, the principal component analysis (PCA) was used to reduce the dimensions of the DataFrame without losing information and still maximizing the interpretation, while the K-Means made data clusters according to the considered conditions.

The algorithms used for the classification were the K-nearest neighbors classifier (KNN), Decision Tree classifier, Random Forest, and Logistic Regressor classifier. Classification algorithms are normally applied in a binary system and so they were used for each result of the adsorption (removal) and desorption (recovery) steps. The classification for the removal was performed according to the remaining REEs present in solution (the lower remaining percentage, the higher the removal). For the recovery, it was the opposite (the higher the percentage of REE in solution, the higher the recovery). The classifications for removal and for recovery were established for every REE, and then the mean value was calculated. Next, the binary classification was assigned depending on the mean value relative to the chosen cut-off value. The process is summarized in Table 2.

The data used for the classification were divided into two sets, a training set (70% of the data), which contained the known output of the assays and was used to train the model. The other set was the test set (30%), which was used to test the model prediction capacity. The stratify option was also used, so both training and test sets had the same percentage of positive cases, which for this analysis would represent high removals and recoveries.

### 3.5. Statistical Analysis

Two-way ANOVA was performed on the removal percentages for the adsorption assays, in which the conditions were compared between each other for the same time points. The desorption results were analyzed using a two-way ANOVA similar to the removal percentage analysis. The conditions’ values were compared between each other at the same time points. All these analyses were conducted using the software Graph Pad Prism version 8.0.2 (Graph Pad Software, Inc., San Diego, CA, USA). A difference was only considered significant when the probability (*p*-value) was lower than 0.05, assuming a 95% confidence interval.

## 4. Conclusions

The column adsorption results confirmed that between 65 and 90% of the total amount of REE was removed, decreasing with the number of cycles. The process included an adsorption step, a desorption step, and an eventual washing before the next cycle. The 3 h desorption steps reached 80% recovery of the entrapped REE after an increase in the initial eluent concentration for all tested REEs. The ML algorithms were successfully applied to the results obtained experimentally to select the best overall operational conditions. They also allowed for the definition of a regression model that estimates any REE adsorption and desorption efficiency, just by using the values of one of the tested REEs. The lack of significant differences between the zeolites tested, Z13X and ZX_NaOH, suggests that the chemical pre-treatment might not be justifiable. Adding to that, the eventual bed washing with NaOH 0.01 M after adsorption–desorption and before another cycle also showed no improvement in the overall efficiency of the process. The application of the optimized conditions leads to an improvement in the desorption step and had an important influence on the following adsorption cycles, improving the overall results obtained by this system to recover REEs from wastewater.

## Figures and Tables

**Table 1:** Column designations for the continuous flow assays.

Column Designation	Zeolite Used	Modification	Washing between Sorption Assays
Z13X_NW	Z13X	Control	Without
Z13X_WW			With
ZNaOH_NW		NaOH 0.1 M	Without
ZNaOH_WW			With

**Table 2:** Binary classification used for each sample evaluated regarding the data from the adsorption (removal) and the desorption (recovery) assays.

Removal (R_m_)		Recovery (R_c_)		Classification Means	Binary Classification
Interval	Classification	Interval	Classification		
80 < R_m_ < 100	1	80 < R_c_ < 100	5	≥3.5	1
60 < R_m_ < 80	2	60 < R_c_ < 80	4		
40 < R_m_ < 60	3	40 < R_c_ < 60	3		
20 < R_m_ < 40	4	20 < R_c_ < 40	2	<3.5	0
00 < R_m_ < 20	5	00 < R_c_ < 20	1		

**Table 3:** Total removal of each REEs for each zeolite tested after 4 cycles.

Removal (%)	La	Ce	Y	Tb	Pr	Eu
Z13X_NW	81.6 ± 6.5	83.3 ± 5.8	80.4 ± 6.7	83.8 ± 6.0	84.7 ± 5.7	83.6 ± 5.5
Z13X_WW	71.9 ± 0.4	74.7 ± 0.2	71.4 ± 2.2	76.2 ± 1.8	76.4 ± 0.8	75.6 ± 2.0
ZNaOH_NW	73.0 ± 3.0	73.6 ± 2.6	83.0 ± 2.0	72.7 ± 2.3	73.2 ± 2.9	72.7 ± 2.5
ZNaOH_WW	68.2 ± 4.1	70.9 ± 4.8	75.1 ± 4.5	72.3 ± 2.4	70.9 ± 3.6	70.7 ± 3.3

**Table 4:** Total recovery percentage of each REE for each zeolite sample tested after 4 cycles.

Recovery (%)	La	Ce	Y	Tb	Pr	Eu
Z13X_NW	9.5 ± 0.1	9.2 ± 0.2	11.0 ± 0.7	10.9 ± 1.1	9.4 ± 0.1	11.5 ± 0.5
Z13X_WW	7.9 ± 1.1	8.4 ± 1.1	10.7 ± 0.6	10.1 ± 0.7	8.3 ± 1.0	11.2 ± 0.6
ZNaOH_NW	11.7 ± 1.7	12.1 ± 2.2	10.5 ± 4.5	13.6 ± 3.5	13.8 ± 2.1	15.8 ± 2.9
ZNaOH_WW	14.9 ± 1.6	13.9 ± 0.8	14.9 ± 1.8	17.5 ± 0.9	16.0 ± 1.3	18.9 ± 1.1

**Table 5:** Resume of the different steps carried out with the solutions initial concentration, flow rates, and duration. Each column had 4 adsorption–desorption cycles, only two of them had a washing in between.

Step	Cycle	Solution	Pump Rate (mL/min)	Duration (h)
Adsorption	1	C_i_ = 60 mg/L for each REE; V_i_ = 5 L	4	72
	2	C_i_ = 10 mg/L for each REE; V_i_ ≈ 5 L		
	3	C_i_ = 60 mg/L for each REE; V_i_ = 5 L		
	4	C_i_ = 25 mg/L for each REE; V_i_ ≈ 5 L		
Desorption	1	1 L of HNO_3_ 0.1 M for each desorption step	8	6
	2			
	3			
	4			
Wash	1	1 L of NaOH 0.01 M for each washing step	15	2
	2			
	3			

## Data Availability

Data will be made available on request.

## Supplementary Materials

The following supporting information can be downloaded at: https://www.mdpi.com/article/10.3390/molecules28247964/s1, Figure S1: Removal over time of the REE by zeolites; Figure S2: Total recovery of the REE from zeolites; Figure S3: Elbow method for PCA and K-Means for the sorption cycles; Figure S4: Elbow method for PCA and K-Means for the desorption cycles; Figure S5: PCA maps; Figure S6: Confusion Matrix; Table S1: ANOVA results for the total adsorption; Table S2: ANOVA results for the total desorption; Table S3: Statistical tests for the desorption difference between each cycle for each tested condition.
