# Supplementary material for: Exploring Optimization of Zeolites as Adsorbents for Rare Earth Elements in Continuous Flow by Machine Learning Techniques
Source: Molecules. 2023 Dec 6;28(24):7964. doi: 10.3390/molecules28247964 (PMC10746106; doi:10.3390/molecules28247964)
Supplement: Supplementary file 1 [file molecules-28-07964-s001.zip › molecules-2711501-supplementary.pdf]

Supplementary material

Exploring Optimization of Zeolites as Adsorbents for Rare Earth Elements in Continuous Flow by Machine Learning Techniques

Óscar Barros [1,2,*], Pier Parpot [1,2], Isabel C. Neves [1,2,*] and Teresa Tavares [1,3]

[1] CEB—Centre of Biological Engineering, University of Minho, Campus de Gualtar, 4710-057 Braga, Portugal; parpot@quimica.uminho.pt (P.P.); ttavares@deb.uminho.pt (T.T.)
[2] CQUM, Centre of Chemistry, Chemistry Department, University of Minho, Campus de Gualtar, 4710-057 Braga, Portugal
[3] LABBELS—Associate Laboratory, 4710-057 Braga, Portugal

1. Results

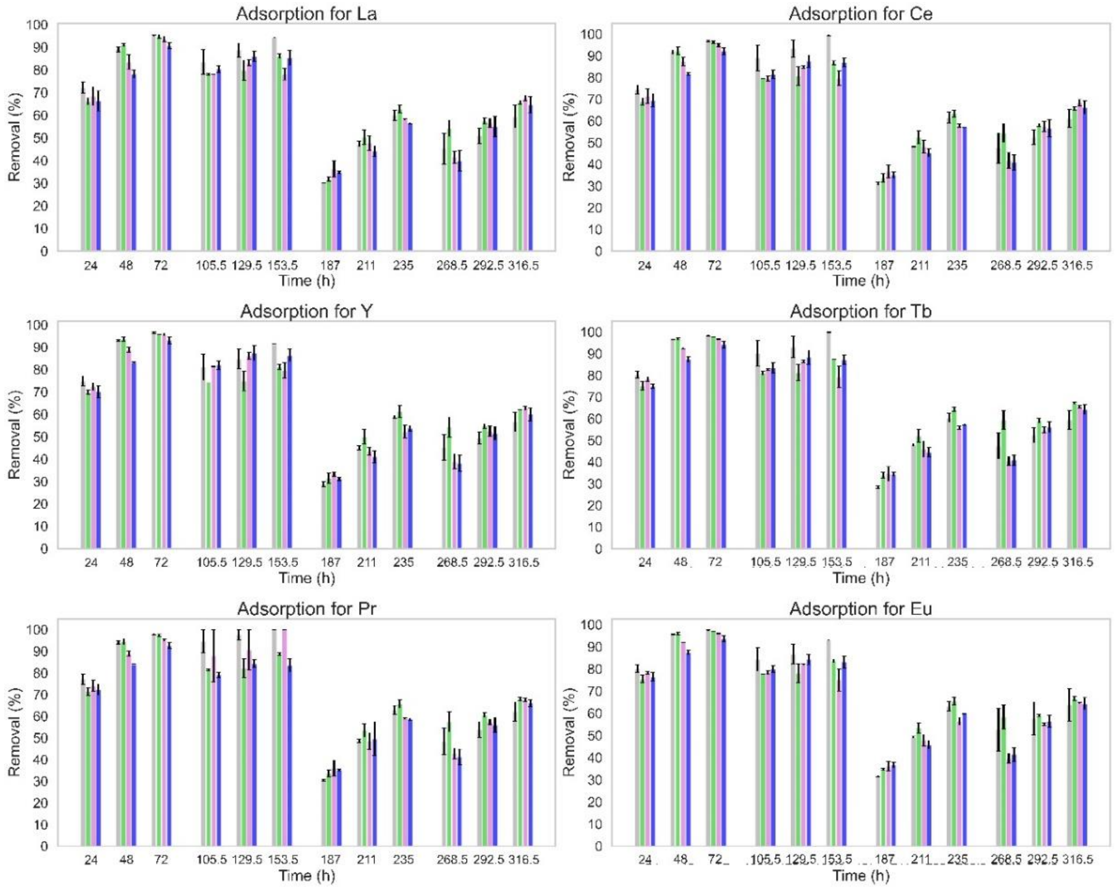

Figure S1: Removal over time for the different REE adsorption for Z13X_NW (grey), Z13X_WW (green), ZNaOH_NW (pink) and ZNaOH_WW (blue). The cycle 1 runs between 24 and 72h, cycle 2 runs between 100.5 and 153.5 h, cycle 3 runs between 187 and 235 h and cycle 4 runs between 268.5 and 316.5 h.

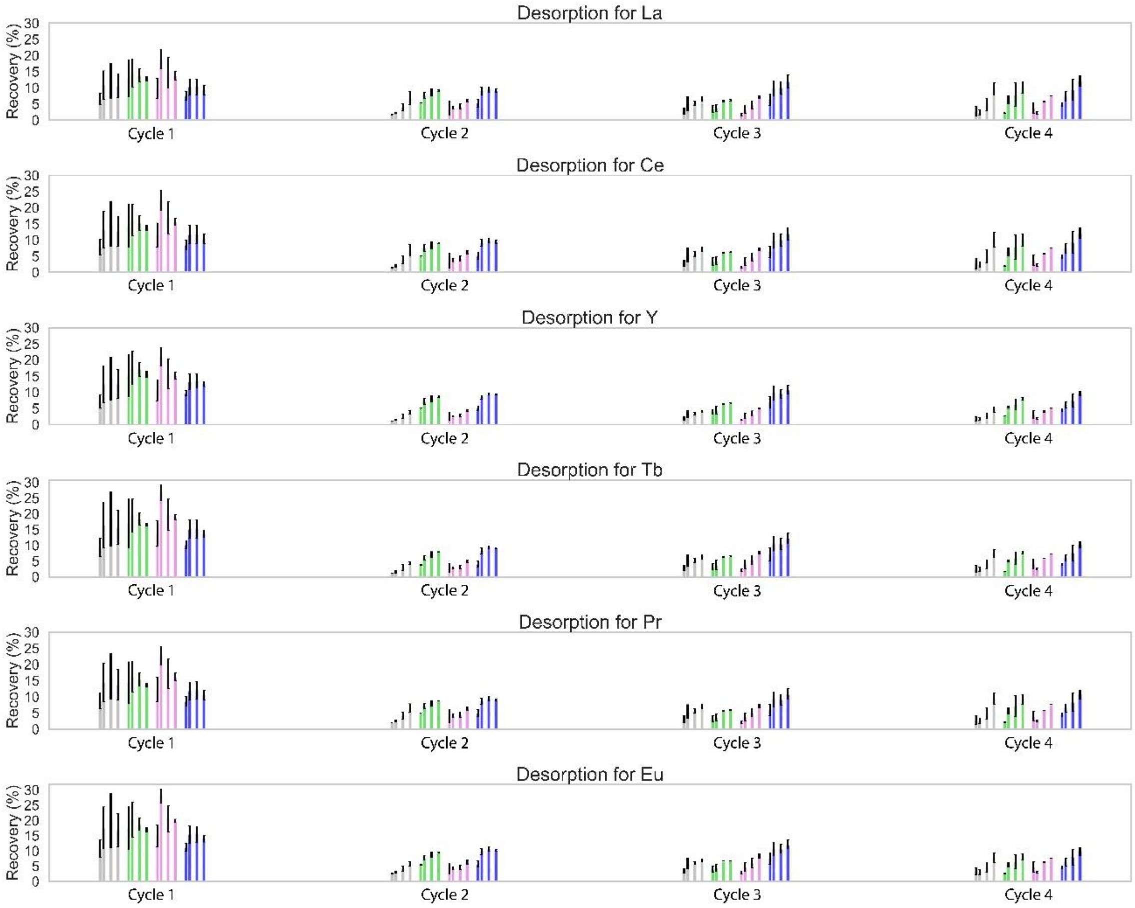

Figure S2: Total recovery for the different REE grouped by cycle and then grouped by sample tested for Z13X_NW ( ), Z13X_WW ( ), ZNaOH_NW ( ) and ZNaOH_NW ( ). Cycle 1 had samples measured at 73.5, 74.5, 76.5 and 78.5 h, cycle 2 includes measurementsat 155, 156, 158 and 160 h. For cycle 3, samples were measured at 236.5, 237.5, 239.5 and 241.5 h and finally cycle 4 includes measurements at 318, 319, 321, 323 h.

1.1. Machine Learning

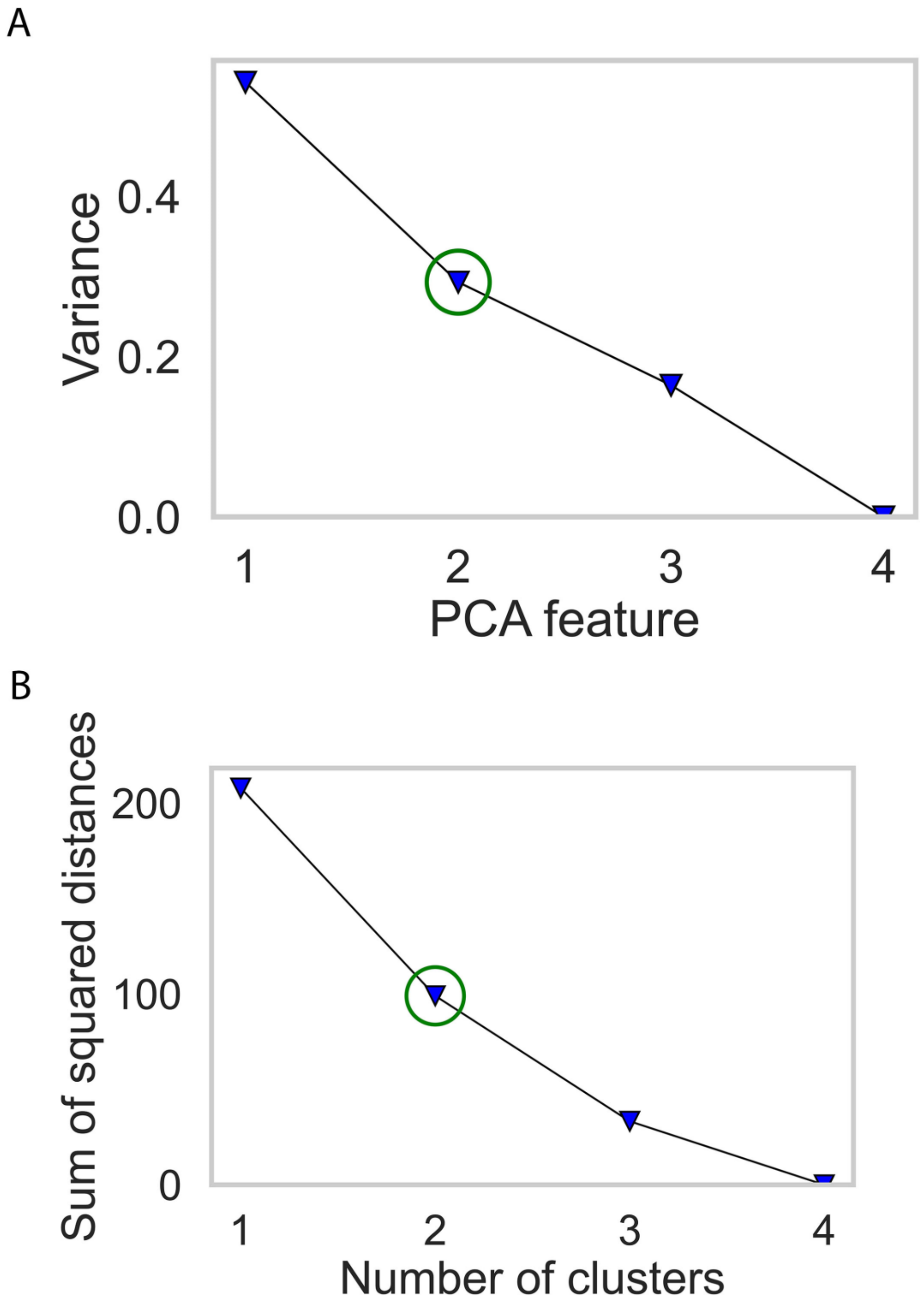

Figure S3: Elbow method for selecting the best option: **A)** PCA and **B)** K-Means for selection of the best condition for multiple adsorption and desorption cycles.

1.2. Adsorption analysis

Table S1: Two-Way ANOVA for the total removal percentage of REE after 4 cycles. The NW refers to the assays without the washing step, and the WW refers to the assays with the NaOH 0.01 M washing step.

Bonferroni's multiple comparisons tests	REE					
	La	Ce	Y	Tb	Pr	Eu
Z13X_NW vs. Z13X_WW	No (ns)	No (ns)	No (ns)	No (ns)	No (ns)	No (ns)
Z13X_NW vs. ZNaOH_NW	No (ns)	No (ns)	No (ns)	No (ns)	No (ns)	No (ns)
Z13X_NW vs. ZNaOH_WW	No (ns)	No (ns)	No (ns)	No (ns)	No (ns)	No (ns)
Z13X_WW vs. ZNaOH_NW	No (ns)	No (ns)	No (ns)	No (ns)	No (ns)	No (ns)
Z13X_WW vs. ZNaOH_WW	No (ns)	No (ns)	No (ns)	No (ns)	No (ns)	No (ns)
ZNaOH_NW vs. ZNaOH_WW	No (ns)	No (ns)	No (ns)	No (ns)	No (ns)	No (ns)

1.3. Desorption analysis

Table S2: Two-Way ANOVA for the total recovery percentage after 4 cycles. The NW refers to the assays without the washing step, and the WW refers to the assays with the NaOH 0.01 M washing step.

Bonferroni's multiple comparisons tests	REE					
	La	Ce	Y	Tb	Pr	Eu
Z13X_NW vs. Z13X_WW	No (ns)	No (ns)	No (ns)	No (ns)	No (ns)	No (ns)
Z13X_NW vs. ZNaOH_NW	No (ns)	No (ns)	No (ns)	No (ns)	No (ns)	No (ns)
Z13X_NW vs. ZNaOH_WW	No (ns)	No (ns)	No (ns)	No (ns)	No (ns)	No (ns)
Z13X_WW vs. ZNaOH_NW	No (ns)	No (ns)	No (ns)	No (ns)	No (ns)	No (ns)
Z13X_WW vs. ZNaOH_WW	No (ns)	No (ns)	No (ns)	No (ns)	No (ns)	No (ns)
ZNaOH_NW vs. ZNaOH_WW	No (ns)	No (ns)	No (ns)	No (ns)	No (ns)	No (ns)

1.4.ML analysis of the desorption optimization

Table S3: Statistical tests performed for all desorption cycles for each REE tested. The Two-Way ANOVA results are related to the comparison with the total REE recovery from the zeolite. A comparison between each cycle for each tested condition was performed. The NW refers to the assays without the washing step and the WW refers to the assays with the NaOH 0.01 M washing step. The optimized cycle is referred to as cycle 5.

REE	Bonferroni's multiple comparisons tests	Two way ANOVA			
		Z13X_NW	Z13X_WW	ZNaOH_NW	ZNaOH_WW
La	Cycle 1 vs. Cycle 2	No (ns)	No (ns)	No (ns)	No (ns)
	Cycle 1 vs. Cycle 3	No (ns)	No (ns)	No (ns)	No (ns)
	Cycle 1 vs. Cycle 4	No (ns)	No (ns)	No (ns)	No (ns)
	Cycle 1 vs. Cycle 5	Yes (****)	Yes (****)	Yes (****)	Yes (****)
	Cycle 2 vs. Cycle 3	No (ns)	No (ns)	No (ns)	No (ns)
	Cycle 2 vs. Cycle 4	No (ns)	No (ns)	No (ns)	No (ns)
	Cycle 2 vs. Cycle 5	Yes (****)	Yes (****)	Yes (****)	Yes (****)
	Cycle 3 vs. Cycle 4	No (ns)	No (ns)	No (ns)	No (ns)
	Cycle 3 vs. Cycle 5	Yes (****)	Yes (****)	Yes (****)	Yes (****)
	Cycle 4 vs. Cycle 5	Yes (****)	Yes (****)	Yes (****)	Yes (****)
Ce	Cycle 1 vs. Cycle 2	No (ns)	No (ns)	No (ns)	No (ns)
	Cycle 1 vs. Cycle 3	No (ns)	No (ns)	No (ns)	No (ns)
	Cycle 1 vs. Cycle 4	No (ns)	No (ns)	No (ns)	No (ns)
	Cycle 1 vs. Cycle 5	Yes (****)	Yes (****)	Yes (****)	Yes (****)
	Cycle 2 vs. Cycle 3	No (ns)	No (ns)	No (ns)	No (ns)
	Cycle 2 vs. Cycle 4	No (ns)	No (ns)	No (ns)	No (ns)
	Cycle 2 vs. Cycle 5	Yes (****)	Yes (****)	Yes (****)	Yes (****)
	Cycle 3 vs. Cycle 4	No (ns)	No (ns)	No (ns)	No (ns)
	Cycle 3 vs. Cycle 5	Yes (****)	Yes (****)	Yes (****)	Yes (****)
	Cycle 4 vs. Cycle 5	Yes (****)	Yes (****)	Yes (****)	Yes (****)
Y	Cycle 1 vs. Cycle 2	No (ns)	No (ns)	No (ns)	No (ns)
	Cycle 1 vs. Cycle 3	No (ns)	No (ns)	No (ns)	No (ns)
	Cycle 1 vs. Cycle 4	No (ns)	No (ns)	No (ns)	No (ns)
	Cycle 1 vs. Cycle 5	Yes (****)	Yes (****)	Yes (****)	Yes (****)
	Cycle 2 vs. Cycle 3	No (ns)	No (ns)	No (ns)	No (ns)
	Cycle 2 vs. Cycle 4	No (ns)	No (ns)	No (ns)	No (ns)
	Cycle 2 vs. Cycle 5	Yes (****)	Yes (****)	Yes (****)	Yes (****)
	Cycle 3 vs. Cycle 4	No (ns)	No (ns)	No (ns)	No (ns)
	Cycle 3 vs. Cycle 5	Yes (****)	Yes (****)	Yes (****)	Yes (****)
	Cycle 4 vs. Cycle 5	Yes (****)	Yes (****)	Yes (****)	Yes (****)
Tb	Cycle 1 vs. Cycle 2	No (ns)	No (ns)	No (ns)	No (ns)
	Cycle 1 vs. Cycle 3	No (ns)	No (ns)	No (ns)	No (ns)
	Cycle 1 vs. Cycle 4	No (ns)	No (ns)	No (ns)	No (ns)
	Cycle 1 vs. Cycle 5	Yes (****)	Yes (****)	Yes (****)	Yes (****)
	Cycle 2 vs. Cycle 3	No (ns)	No (ns)	No (ns)	No (ns)
	Cycle 2 vs. Cycle 4	No (ns)	No (ns)	No (ns)	No (ns)
	Cycle 2 vs. Cycle 5	Yes (****)	Yes (****)	Yes (****)	Yes (****)
	Cycle 3 vs. Cycle 4	No (ns)	No (ns)	No (ns)	No (ns)
	Cycle 3 vs. Cycle 5	Yes (****)	Yes (****)	Yes (****)	Yes (****)

	Cycle 4 vs. Cycle 5	Yes (****)	Yes (****)	Yes (****)	Yes (****)
Pr	Cycle 1 vs. Cycle 2	No (ns)	No (ns)	No (ns)	No (ns)
	Cycle 1 vs. Cycle 3	No (ns)	No (ns)	No (ns)	No (ns)
	Cycle 1 vs. Cycle 4	No (ns)	No (ns)	No (ns)	No (ns)
	Cycle 1 vs. Cycle 5	Yes (****)	Yes (****)	Yes (****)	Yes (****)
	Cycle 2 vs. Cycle 3	No (ns)	No (ns)	No (ns)	No (ns)
	Cycle 2 vs. Cycle 4	No (ns)	No (ns)	No (ns)	No (ns)
	Cycle 2 vs. Cycle 5	Yes (****)	Yes (****)	Yes (****)	Yes (****)
	Cycle 3 vs. Cycle 4	No (ns)	No (ns)	No (ns)	No (ns)
	Cycle 3 vs. Cycle 5	Yes (****)	Yes (****)	Yes (****)	Yes (****)
	Cycle 4 vs. Cycle 5	Yes (****)	Yes (****)	Yes (****)	Yes (****)
Eu	Cycle 1 vs. Cycle 2	No (ns)	No (ns)	No (ns)	No (ns)
	Cycle 1 vs. Cycle 3	No (ns)	No (ns)	No (ns)	No (ns)
	Cycle 1 vs. Cycle 4	No (ns)	No (ns)	No (ns)	No (ns)
	Cycle 1 vs. Cycle 5	Yes (****)	Yes (****)	Yes (****)	Yes (****)
	Cycle 2 vs. Cycle 3	No (ns)	No (ns)	No (ns)	No (ns)
	Cycle 2 vs. Cycle 4	No (ns)	No (ns)	No (ns)	No (ns)
	Cycle 2 vs. Cycle 5	Yes (****)	Yes (****)	Yes (****)	Yes (****)
	Cycle 3 vs. Cycle 4	No (ns)	No (ns)	No (ns)	No (ns)
	Cycle 3 vs. Cycle 5	Yes (****)	Yes (****)	Yes (****)	Yes (****)
	Cycle 4 vs. Cycle 5	Yes (****)	Yes (****)	Yes (****)	Yes (****)

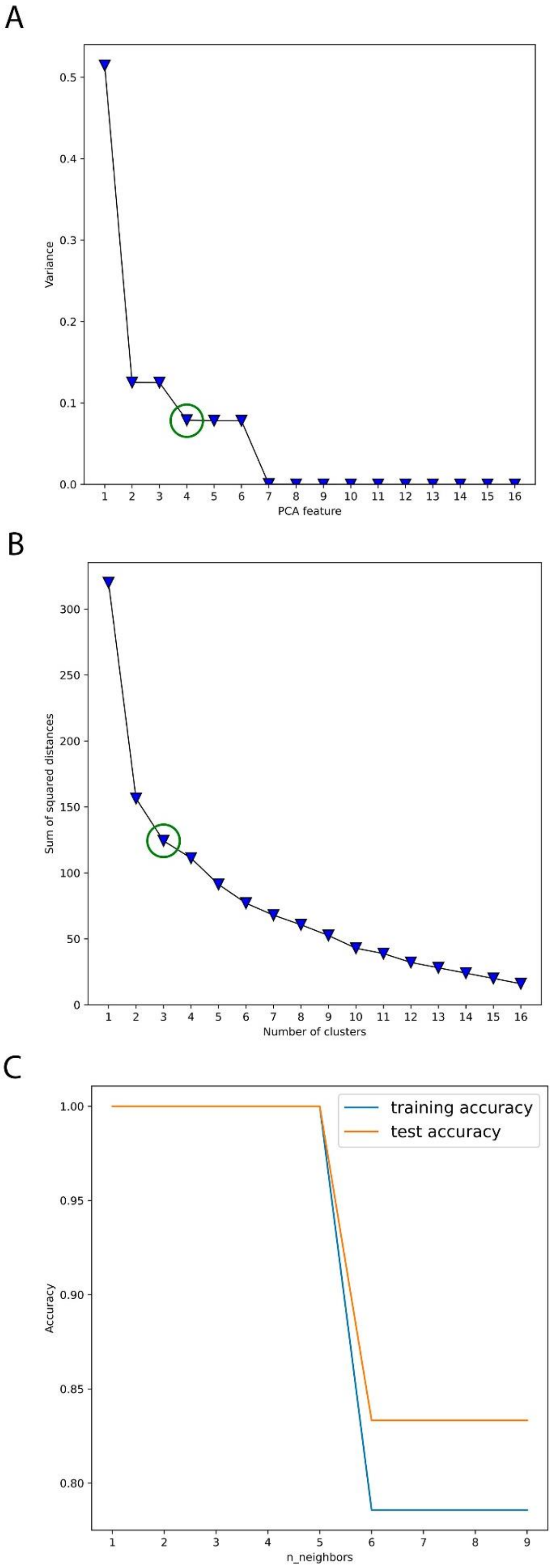

Figure S4: Elbow method for selecting the best option: **A)** PCA, **B)** K-Means and **C)** KNN Classifier for the comparison of the desorption cycles results with the optimized cycle.

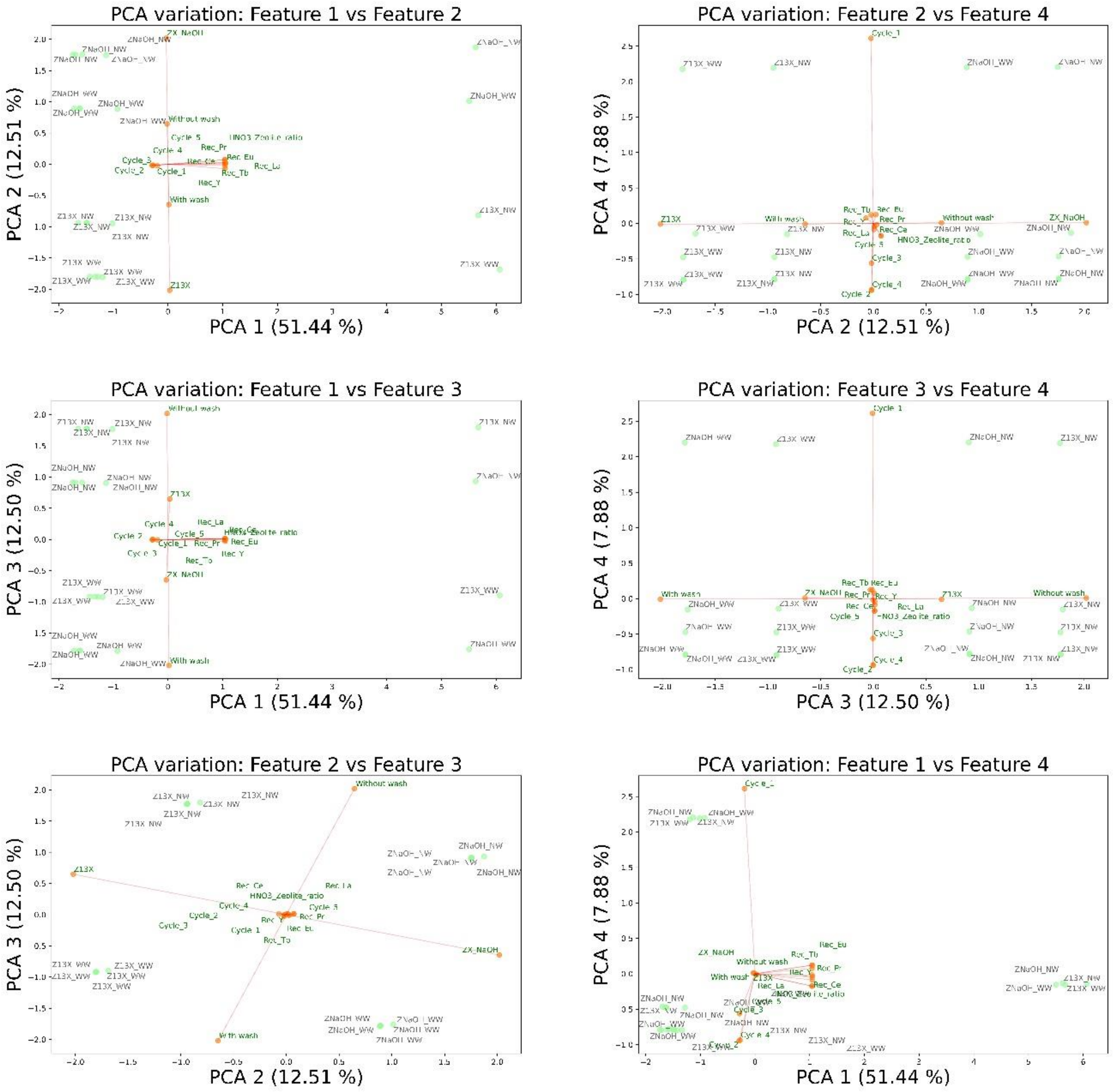

Figure S5: PCA maps. Since more than two features were selected, each map represents the distinct distribution for the selected features.

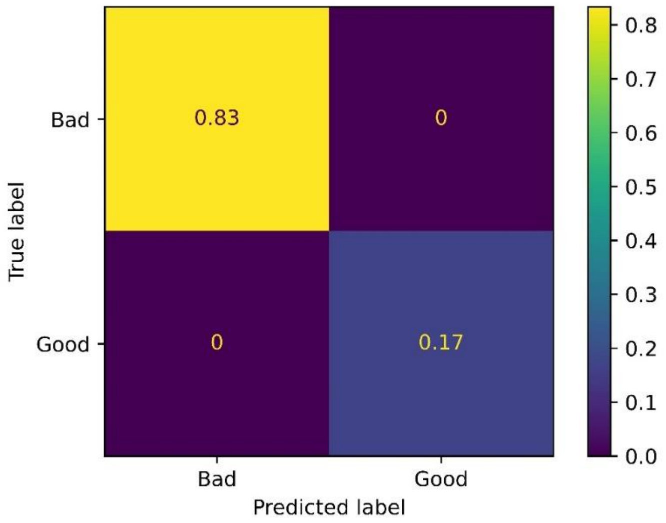

Figure S6: Graphical representation of the confusion matrix for all tested classifiers
